# X-Linked *MTMR8* Diversity and Evolutionary History of Sub-Saharan Populations

**DOI:** 10.1371/journal.pone.0080710

**Published:** 2013-11-25

**Authors:** Damian Labuda, Vania Yotova, Jean-François Lefebvre, Claudia Moreau, Gerd Utermann, Scott M. Williams

**Affiliations:** 1 Centre de Recherche, CHU Sainte-Justine, Université de Montréal, Montréal, Québec, Canada; 2 Département de Pédiatrie, Université de Montréal, Montréal, Québec, Canada; 3 Department of Medical Genetics, Molecular and Clinical Pharmacology, Medical University of Innsbruck, Innsbruck, Austria; 4 Department of Genetics, Geisel School of Medicine, Dartmouth College, Hanover, New Hampshire, United States of America; Universitat Pompeu Fabra, Spain

## Abstract

The genetic diversity within an 11 kb segment of the *MTMR8* gene in a sample of 111 sub-Saharan and 49 non-African X chromosomes was investigated to assess the early evolutionary history of sub-Saharan Africans and the out-of-Africa expansion. The analyses revealed a complex genetic structure of the Africans that contributed to the emergence of modern humans. We observed partitioning of two thirds of old lineages among southern, west/central and east African populations indicating ancient population stratification predating the out of Africa migration. Age estimates of these lineages, older than coalescence times of uniparentally inherited markers, raise the question whether contemporary humans originated from a single population or as an amalgamation of different populations separated by years of independent evolution, thus suggesting a greater antiquity of our species than generally assumed. While the oldest sub-Saharan lineages, ∼500 thousand years, are found among Khoe-San from southern-Africa, a distinct haplotype found among Biaka is likely due to admixture from an even older population. An East African population that gave rise to non-Africans underwent a selective sweep affecting the subcentromeric region where *MTMR8* is located. This and similar sweeps in four other regions of the X chromosome, documented in the literature, effectively reduced genetic diversity of non-African chromosomes and therefore may have exacerbated the effect of the demographic bottleneck usually ascribed to the out of Africa migration. Our data is suggestive, however, that a bottleneck, occurred in Africa before range expansion.

## Introduction

In light of recent data, human evolutionary history looks much more complex than what geneticists postulated only a decade ago [1]. For example, in addition to new evidence of archaic admixture outside Africa [2]–[4], the time scale of human evolution needs to be extended. The generation span of humans and their ancestors appears longer than was previously estimated, and an older age of independent human lineages that collectively contributed to the modern genome was documented [5]–[10]. Nevertheless, the greatest genetic diversity among human populations is observed in sub-Saharan Africa, which leads to revisiting the old question [11], [12]:

Is greater genetic diversity in Africa due only to older and larger ancestral African population sizes as compared to those outside of Africa, or does it reflect the impact primarily of the out of Africa population bottleneck(s) in reducing the genetic diversity outside Africa? If at the time of out-of-Africa migration Africans and migrant populations did not differ in their genetic structure, but African populations stayed larger than non-Africans, greater African diversity would be expected to result from an accumulation of new low frequency local variants. In contrast, a restricted sampling of alleles due to a stringent out-of-Africa bottleneck [13] would cause a relative paucity of the genetic diversity in non-Africans [14]. Both above scenarios turned out to be too simplistic. New findings have provided support for the idea that genetic diversity of non-Africans was additionally enriched by admixture with Neandertals [2], [4] and Denisovans [3]. In Africa, low frequency derived alleles in the dys44 segment are spread on different haplotypes, which implies substantial number of recombinations and gene conversions, and thus long evolutionary time since the corresponding mutations have occurred [10]. Such alleles are necessarily ancient and their limited occurrence suggested archaic admixture within Africa itself, supported by additional evidence [9], [15]–[18]. Although extensive gene flow shaped the diversity of sub-Saharan Africans over various periods [19]–[22], traces of the ancestral subdivisions can still be recognized in the genetic record [6], [20], [23]–[26]; the homogenizing effect of gene flow that in general will partially conceal the record of the ancestral population structure, is expected to be less obvious in areas of low recombination and strong linkage disequilibrium [27]. Studying loci with such characteristics is therefore of great significance in terms of unravelling human population histories. In depth analyses of single autosomal and X-linked loci complement studies of uniparentally inherited mitogenomes and Y-chromosomes with their well resolved genealogies. However, the time depth of autosomal or X-linked genealogies is roughly four and three-times deeper, respectively, than that of the genealogies of the uniparentally inherited loci. The “whole genome studies” amalgamate information from individual loci to provide a synthetic overall view of the human genome history. In contrast, single locus oriented studies, can reveal particular and sometimes unusually important historical events, such as that of archaic admixture within Africa, and/or that of Neandertal admixture outside Africa, as documented in our earlier studies focusing on specific DNA segments [4], [6], [10], [28], [29].

Here we studied an 11 kb DNA segment with low-recombination frequencies [30] located in the Xq11.1 genomic region. This segment is located within the myotubularin related protein 8 gene (*MTMR8*), in the centromeric portion of the X chromosome, earlier found to have reduced sequence diversity in non-African populations [31]–[33]. Our study showed that *MTMR8* diversity in sub-Saharan Africans can reveal much about the evolutionary history of these populations. Specifically, ancient population structure in the geographic partitioning reflects separate evolutionary histories of the southern, western/central and the eastern African clades. Our results are consistent with the enrichment of the African diversity through fragmentation of its populations over long periods of their history as well as by admixture among archaic populations. They also support the hypothesis of an early bottleneck in out of Africa migrants, prior to their range expansion outside of Africa.

## Materials and Methods

### DNA samples

A total of 160 X chromosomes were analyzed, 73 drawn from DNA samples of the Human Genome Diversity Panel of Centre d'Étude du Polymorphisme Humain, CEPH-HGDP [34]: Bantu speakers NE, Kenya (n = 10); Biaka Pygmy, Central African Republic (n = 13); San, Namibia (n = 7); Mandenka, Senegal (n = 13); M'Buti Pygmy, Democratic Republic of Congo (n = 13); Yoruba, Nigeria (n = 17). The remaining samples were from earlier studies (see [4], [6], [35], [36]), and included Gabonese (n = 12); Khoe-San, collected in Smithdrift, South Africa (n = 11); Ethiopians (n = 15); Europeans (n = 13; 2 Bulgarians, 3 French, 2 French-Canadians, 2 Germans, 4 Italians), South East Asians (n = 14; 6 Chinese, 4 Vietnamese, 2 Filipinos, 2 Indians); Near East/North Africa (n = 12; 2 Iran, 4 Lebanon, 4 Egypt, 2 Morocco); Native Americans (n = 10; 6 Chipewyans, 4 Maya) (see table S1). They were obtained from the collaborating academic institutions or were collected by us, following a protocol approved by the Institutional Ethics Committee at the Centre Hospitalier Universitaire Sainte-Justine. Common chimpanzee DNA was extracted from a peripheral blood sample provided by Clément Lanthier from Granby Zoo (Québec), taken during the course of routine veterinary care. All Sub-Saharan African chromosomes (n = 111; 107 male and 2 female samples) and 7 non-African chromosomes (3 females and 1 male sample) were analyzed directly by resequencing; the remaining 42 non-African chromosomes were screened for additional polymorphisms by heteroduplex analysis followed by direct sequencing of the fragments of interest. Note that samples from Egypt and Morocco are counted among non-Africans. In addition, we used the genomic diversity data available online for HGDP and HapMap3 DNA populations [34], [37]–[39].

### Detection of sequence polymorphism

PCR-primers were designed to amplify 21 overlapping fragments covering an 11024 bp-long segment of *MTMR8* (NT011669), starting in intron 3 and ending in intron 5 (table S2). Amplifications were carried out in 20 µl using 5 ng DNA, 0.75 U of Platinum® Taq DNA polymerase (Invitrogen Canada Inc.) in the Platinum® PCR buffer containing 1.5 mM MgCl_2_, 0.2 mM each dNTP, and 2 µM each primer. Reaction started with 5 min incubation at 94°C followed by 35 cycles of 30 s at 94°C, 30 s at 55°C (except for fragment 9 at 61°) and 30 s at 72°C, to end in 10 min incubation at 72°C. Both strands of the products were sequenced on ABI 3730 DNA sequencer. In addition to 118 chromosomes analyzed in this way we screened 21 female non-African samples (Asia, Europe, and Native America - 5 from each group, and 6 from Near East/North Africa) for the presence of polymorphisms by DNA temperature-melting heteroduplex-detecting analysis [40]. It was carried out in HR-1 High Resolution Melter, Idaho Technology, using LCGreen I Melting Dye in standard Roche LightCycler® glass capillary tubes. We used previously detected polymorphisms to create heterozygous samples serving as positive controls for heteroduplex detection in the analyzed DNA fragments. Two heteroduplexes were detected in non-African samples, adding two new haplotypes. By sequencing they were found to be due to a C>T transition in position 1890874 (mutation 32) and to a T>C polymorphism at position 1887715 (mutation 19), already known from resequencing of the African samples (table 1).

**Table 1:** *MTMR8* segment haplotypes.

SNPs ID	this study	this study	this study	this study	this study	this study	rs6624109	this study	this study	this study	rs5964355	rs5964767	this study	this study	this study	rs6653194	this study	this study	rs5964768	this study	rs5964769	rs17301157	this study	this study	rs1883667		this study	this study	this study	this study	this study	this study		this study	this study	rs2143485	this study	rs5964770	this study	Populations									Counts
Human Feb. 2009 (GRCh37/hg19) assembly	63565561	63565562	63565568	63565691	63565818	63566179	63566648	63566740	63566864	63567014	63567951	63568366	63568754	63568859	63569203	63569307	63569355	63569593	63569727	63569969	63569979	63570024	63571405	63571438	63571569		63571733	63571753	63571974	63572010	63572288	63572723		63572886	63573344	63573673	63573863	63575039	63575448	Bantu NE	Bia	Eth	Gabon	Khoe-San	Mandenka	Mbuti	Yoruba	Non Africans	Total
network mut. positions	1–2		3	4	5	6	7	8	9	10	11	12	13	14	15	16	17	18	19	20	21	22	23	24	25	25*	26	27	28	29	30	31	31*	32	33	34	35	36	37										
Ancestral	G	A	C	C	G	G	G	C	A	A	C	A	T	C	T	C	A	A	T	A	A	G	G	A	G	G	G	G	C	A	A	G	G	C	C	G	C	A	T										
Neandertal	.	.	.	.	.	A	.	.	.	.	.	.	.	.	.	.	.	.	.	.	.	.	.	.	.		.	.	.	.	.	.		.	.	.	.	.	.										
Denisova	.	.	.	.	.	.	.	.	.	.	.	.	.	.	.	.	.	.	.	.	.	N	.	.	.	.	.	.	.	.	.	.	.	.	.	.	.	.	.										
H 1	A	G	A	.	.	A	.	.	.	.	.	.	.	.	.	.	.	.	.	.	T	.	.	.	A	.	.	.	.	.	.	.	.	.	.	.	.	.	.	4	5	3	2	3	6	8	7		38
H 2	A	G	A	.	.	A	.	.	.	.	.	.	.	.	.	.	.	.	.	.	T	.	.	.	A	.	.	.	.	.	.	.	A	.	.	.	.	.	.							1			1
H 3	A	G	A	.	.	A	.	.	.	.	.	.	.	.	.	.	.	.	.	.	T	.	.	.	A	.	.	.	.	C	.	.	.	.	.	.	.	.	.	1					1				2
H 4	A	G	A	.	.	A	.	.	.	.	.	.	.	.	.	.	.	.	.	.	T	.	.	.	.	.	.	.	.	.	.	.	.	.	.	.	.	.	.		1						2		3
H 5	A	G	A	.	.	A	.	.	.	.	.	.	.	.	.	.	.	.	.	.	.	.	.	.	.	A	.	.	.	.	.	.	.	.	.	.	.	.	.					2					2
H 6	A	G	A	.	.	A	.	.	.	.	.	.	.	.	.	.	.	.	.	T	T	.	.	.	A	.	.	.	.	C	.	.	.	.	.	.	.	.	.								1		1
H 7	A	G	A	.	.	A	.	.	.	.	.	.	.	.	.	.	T	.	.	.	T	.	.	.	A	.	.	.	.	.	.	.	.	.	.	.	.	.	.					1					1
H 8	A	G	A	.	.	A	.	.	.	.	.	.	.	.	C	.	.	.	.	.	T	.	.	.	.	.	.	.	.	.	.	.	.	.	.	.	.	.	.	1			3			1			5
H 9	A	G	A	.	.	A	.	.	.	.	.	.	.	T	.	T	.	.	.	.	.	.	.	.	.	.	.	.	.	.	.	A	.	.	.	.	T	.	.	1	4	1	5		4		5		20
H 10	A	G	A	.	.	A	.	.	.	.	.	.	A	.	.	.	.	.	.	.	T	.	.	.	A	.	.	.	.	.	.	.	.	.	.	.	.	.	.	1			1		2	2			6
H 11	A	G	A	.	.	A	.	.	.	T	.	.	.	.	.	.	.	.	.	.	T	.	.	.	A	.	.	.	.	.	.	.	.	.	T	.	.	.	.								1		1
H 23	A	G	A	.	.	A	T	.	.	.	G	G	.	.	.	.	.	.	.	.	.	.	.	.	.	.	.	.	.	.	.	.	.	.	.	T	.	G	.									1	1
H 24	A	G	A	.	.	A	T	.	.	.	G	G	.	.	.	.	.	.	C	.	.	.	.	.	.	.	.	.	.	.	.	.	.	T	.	T	.	G	.									1	1
H 12	A	G	A	.	.	A	T	.	.	.	G	G	.	.	.	.	.	.	C	.	.	.	.	.	.	.	.	.	.	.	.	.	.	.	.	T	.	G	.	1		8						46	55
H 13	A	G	A	.	.	A	T	.	.	.	G	G	.	.	.	.	.	.	C	.	.	.	.	.	.	.	A	.	.	.	.	.	.	.	.	T	.	G	.			1							1
H 14	A	G	A	.	.	A	T	.	.	.	G	G	.	.	.	.	.	.	C	.	.	.	A	.	.	.	.	.	.	.	.	.	.	.	.	T	.	G	.									1	1
H 15	A	G	A	.	.	A	T	.	.	.	G	G	.	.	.	.	.	.	C	.	.	A	.	.	.	.	.	.	.	.	.	.	.	.	.	T	.	G	.			1							1
H 16	A	G	A	T	.	A	T	.	.	.	G	G	.	.	.	.	.	.	C	.	.	A	.	.	.	.	.	.	.	.	.	.	.	.	.	T	.	G	.			1		1					2
H 17	.	.	.	.	.	A	.	.	.	.	.	.	.	.	.	.	.	.	.	.	.	.	.	.	.	.	.	.	T	.	.	.	.	.	.	.	.	.	.	1			1	7		1			10
H 18	.	.	.	.	.	A	.	.	.	.	.	.	.	.	.	.	.	.	.	.	.	.	.	C	.	.	.	.	T	.	.	.	.	.	.	.	.	.	.								1		1
H 19	.	.	.	.	.	A	.	G	.	.	.	.	.	.	.	.	.	.	.	.	.	.	.	.	.	.	.	.	.	.	.	.	.	.	.	.	.	.	G					2					2
H 20	.	.	.	.	.	A	.	G	.	.	.	.	.	.	.	.	.	.	.	.	.	.	.	.	.	.	.	.	.	.	G	.	.	.	.	.	.	.	G					1					1
H 21	.	.	.	.	.	A	.	G	.	.	.	.	.	.	.	.	.	.	.	.	.	.	.	.	.	.	.	A	.	.	G	.	.	.	.	.	.	.	G					1					1
H 22	.	.	.	.	T	.	.	.	T	.	.	.	.	.	.	.	.	G	.	.	.	.	.	.	.	.	.	.	.	.	.	.	.	.	.	.	.	.	.		3								3
n																																								10	13	15	12	18	13	13	17	49	160

The haplotype spans 11 Kb of the *MTMR8* gene, starting in intron 3 and ending in intron 5; the location of its polymorphic sites within the hg 19 genome reference sequence are shown in the third line. New alleles appear on the background of ancestral (chimpanzee) alleles, which are also shared with Neandertal and Denisova sequences, except for the polymorphic site 6 (highlighted in grey) where the derived allele is the same as that found in the Neandertal genome. The polymorphic sites 25 and 31, involving CpG-dinucleotides, are assumed to have mutated twice, indicated by asterisk, to create separate haplotypes 5 and 2, respectively (both found among Khoe-San).

### Haplotypes and network

The ancestral alleles of the MTMR8 polymorphisms were inferred by comparison with the primate outgroup sequences of chimpanzee, orangutan and macaque. The human allele identical by state with a chimpanzee or at least two other outgroup sequences was considered ancestral. All distinct haplotypes could have been derived from the data without any ambiguity because all but two African samples represented hemizygous males and all females were either homozygous or when heterozygous it was only in a single haplotype position (table 1). The haplotype tree reduces to a “perfect phylogeny” tree after excluding derived allele A at the site 1889557 (mutation 25) in haplotype 5 (seen in two copies in Khoe-San), and derived allele A at the site 1890711 (mutation 31) in haplotype 2 (a single copy in M'Buti). These two particular polymorphisms are due to G>A transitions within hypermutable CpG-dinucleotides [41] thereby providing limited phylogenetic information. We assumed that haplotypes 2 and 5 were due to independent recurrent transitions (coded as mutation 25* and 31*, respectively) rather than resulting from an unlikely sequence of recombination events in this genomic region of particularly low recombination activity [30].

### Statistical analysis

Coalescence analysis was carried out according to the method of Griffiths and Tavaré [42] using *genetree*, version 9.0, on the full data set (n = 160), on all Sub-Saharan Africans together (n = 111), their eight subpopulations, and Sub-Saharan Africans +1 (n = 112) by addition of haplotype 23, found in a single copy in Lebanon but possibly of African origin given its position in the network. Maximum likelihood estimates of Θ (i.e. Θ*_ML_*; see list of abbreviations in Material S1), the time to the most recent common ancestor (TMRCA) and the age of mutations were obtained conditional on the haplotype tree, assuming an infinite-sites model, random mating and constant population size or exponential growth. The number of iterations per run was sufficiently large for the results to remain constant over repeated runs differing only in the random seed number. Estimates of *Θ_ML_* made with a model of exponential growth concurrently yielded an estimate of the growth rate *β*. Particularly, in this model the population size exponentially declines backward in time at rate *β* from a current size *N_(0)_* (or 1.5 *N_(0)_* chromosomes), such that the size of the population at time *t* is *N_(t)_ =  N_(0)_ e^−βt^* (note that *t* = *g*/*1.5N_(0)_*, where *g* is the number of generations ago). Using *genetree* we jointly estimated Θ*_ML_* and the growth parameter *β*. First, we explored different values of *β* at fixed Θ*_ML_* (the starting values were those obtained under a constant population size scenario). Subsequently, for the selected *β*, we explored the likelihood density of Θ, to finally obtain the maximum likelihood estimates of both parameters after few rounds of such simulations. The ρ-statistics [43] that evaluates the average number of mutations till coalescence was calculated using Network 4.5.0.2 software [44]. This statistic [45], [46] is equivalent to Thomson approach [47], [48]. The substitution rate in the 11024 bp *MTMR8* segment was estimated at 1.49×10^−4^ per generation (or 1.35×10^−8^ per bp per generation) from the human-chimpanzee divergence of 0.0081 (±0.0009) per bp, assuming a separation time of 7.5 million years (My) and an average generation time along both lineages of 25 years [7], [49]. The corresponding autosomal rate can be evaluated by multiplying by 3(α+1)/2(α+2), where α is the ratio of male to female mutation rate [50], [51].

ARLEQUIN software, v. 3.1 [52] was employed to compute different population statistics (haplotype count *k* and diversity *G*, count of segregating sites *S* and nucleotide diversity as well as different estimates of the scaled mutation parameter Θ, global and pairwise F_ST_) and to carry out neutrality tests such as Tajima's *D* [53] and Fu's *F_S_* [54], as well as tests according to Ewens and Watterson [55], [56], Slatkin [57], and Chakraborty's population amalgamation test [58]. For detailed description of the parameters and tests please refer to the Arlequin manual [52]. We also used DnaSP software v.5 [59] to estimate Θ*_H_* and carry out the Fay & Wu H test [60].

### Coalescence simulations

Simulations were performed using the msHot software [61], a modification of the ms program [62]. They were used to evaluate the effect of demography on the estimates of Θ*_ML_* [42], Θ*_S_* [63] and Θ*_П_* [53] under a simple version of the standard neutral model at constant population size, at population growth, demographic bottleneck and at population subdivision.

## Results

Table 1 presents sequence diversity of the *MTMR8* segment in a worldwide sample of 160 X chromosomes. Out of 24 haplotypes, 21 are observed in sub-Saharan Africa and only 4 are found outside of Africa (table 2). Furthermore, three of the non-African haplotypes are singletons. This dearth of diversity in non-Africans is also reflected in their very low nucleotide diversity (π = 0.011x10^−3^) and all other summary statistics (table 2 and table S3). In contrast, the nucleotide diversity of sub-Saharan Africans (π = 0.46×10^−3^) falls within the range observed at other X-linked segments [11], [28], [64], [65]. Neutrality tests [53]–[56], [60] are consistent with neutrality of *MTMR8* in sub-Saharan Africa (Table S3). Therefore, this locus is well suited to infer population history of sub-Saharan Africans. In non-African populations the situation is opposite, tests indicate a non-neutral evolution (Table S3) and the virtual absence of common sequence polymorphisms (table 1 and [32], [33]) what renders *MTMR8* uninformative for population history inferences. Therefore, there was no reason to extend the sample size of non-Africans and we have focused our analysis on sub-Saharan populations.

**Table 2:** Sub-Saharan African and non-African Diversity and Neutrality Tests (all abbreviations are listed in Material S1).

	Sub Saharan Africa	Non-Africans	Total
**Summary Statistics**			
***n***	111	49	160
***k***	21	4	24
***S***	34	3	36
Θ***_П_***	5.04	0.12	5.82
Θ***_H_***	6.13	1.96	7.50
Θ***s***	6.45	0.67	6.37
**Hom = (1-** ***G*** **)_obs_**	0.16	0.88	0.19
**Ewens-Watterson**			
**Hom = (1-** ***G*** **)_exp_**	0.12	0.52	0.12
**Watterson**'**s ** ***p***	0.914	1.000	0.964
**Slatkin**'**s ** ***p***	0.936	1.000	0.996
**Chakraborty**'**s**			
***k*** **_exp_**	16.29	1.52	19.99
***P*** ** (** ***k*** ** or more haplotypes)**	0.025	0.008	0.001
**Tajima**'**s**			
***D***	−0.66	−1.70	−0.26
***p***	0.264	0.012	0.415
**Fu**'**s**			
***k*** **_exp_**	16.29	1.52	19.99
***Fs***	−2.15	−4.26	−1.58
***p***	0.292	0.000	0.381
**Fay & Wu**'**s ** ***H***			
***H***	−2.24	−1.84*	−3.08
**Normalized ** ***H***	−0.492	−2.51	−0.680
***p***	0.174	0.005	0.144

* the ***H*** statistics of −**9.44** (*p*<0.001) is obtained for a combined population of non-Africans and Ethiopians (Θ*_π_* = 1.24; Θ*_H_* = 10.6.

**Figure 1:**
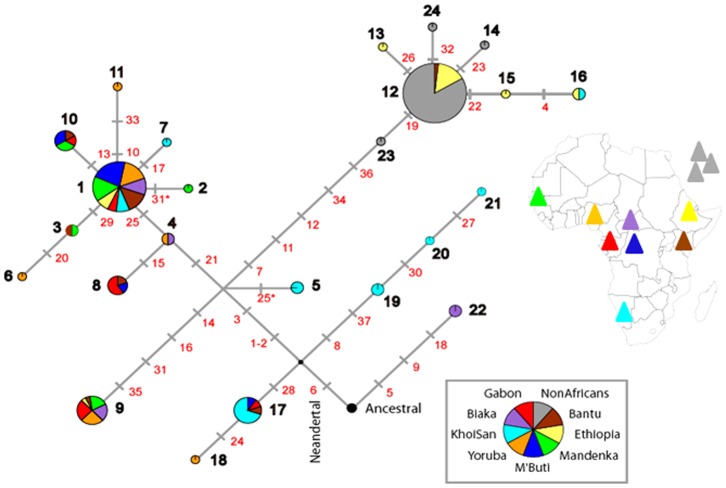
Network of MTMR8 haplotypes. Haplotype frequencies are proportional to the surface of the circle (or to its single colored segment within a population group). Numbering of mutations and haplotypes is the same as in table 1. Asterisks indicate two mutations in the CpG-sites 25 and 31 that presumably represent independent substitutions leading to separate haplotypes 5 and 2, respectively (both found among Khoe-San). When a series of mutation occurs on a single branch their order of appearance is arbitrary as we cannot know which one was first or last based on the presented data.

**Figure 2:**
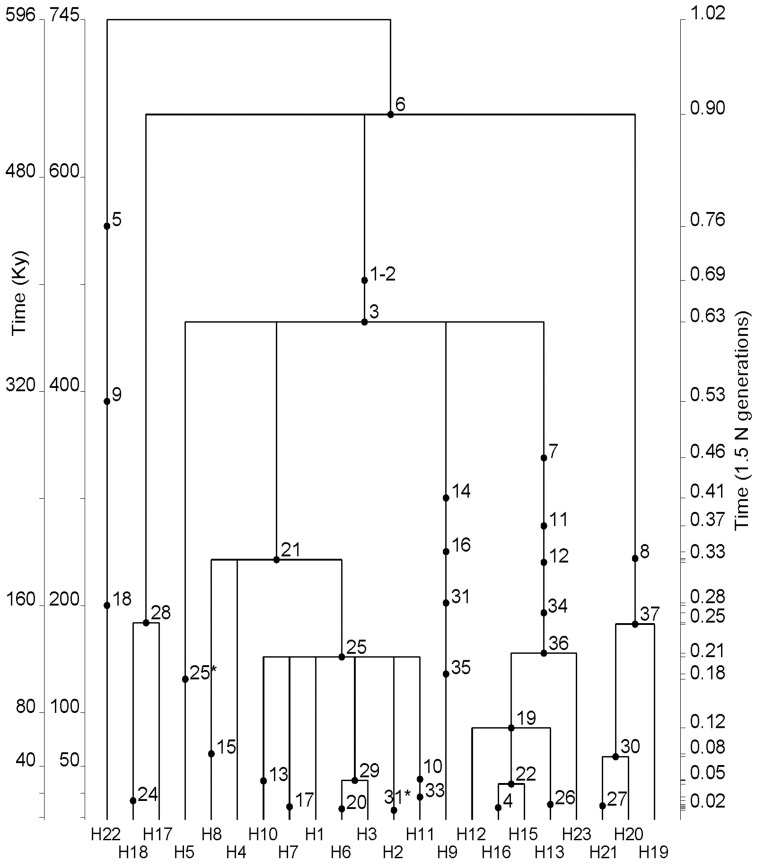
Coalesecent analysis of the MTMR8 tree in Sub-Saharan Africa. The time scale in thousands of years is calculated using 7.5 My (internal left scale) or 6 My (external left scale) of sequence divergence between human and chimpanzee lineages. Numbering of mutations and haplotypes is the same as in fig. 1 and table 1. Note that a Levantine chromosome carrying haplotype 23 was included in this analysis.

The *MTMR8* haplotypes (table 1), considering haplotype 22 apart, form a simple network with the oldest branches separating Southern, Western/Central and Eastern African lineages (fig. 1). The TMRCA was obtained by *genetree* [42] under a modest demographic growth in Africa (***β*** = 0.8) with Θ*_ML_* = 8.7 (*N_(0)ML_* = 19,500) (see Materials and Methods). Non-African samples were excluded from this analysis except for a single Lebanese chromosome carrying haplotype H23 because of its proximity to Africa and likely sub-Saharan origin (figs. 1 and 2). TMRCA was estimated at 29 800±7 000 generations corresponding to 745±175 thousand years (Ky) (fig. 2 and table 3). It falls within the time range of sequence divergence of Neandertals, Denisovans and modern humans [7], [66] consistent with the derived allele A at the site 1884167 (mutation 6) being shared with Neandertals [2]. Interestingly, Denisova genome still carry the ancestral G, but the derived A shows up again in the newest high coverage Altai Neandertal sequence (http://www.eva.mpg.de/neandertal/index.html). In Biaka Pygmies from the Central African Republic we find a structurally distinct, ancient haplotype H22, whose lineage originates directly from the root of the tree (fig. 1). Given its characteristics we propose it as a plausible candidate to represent an archaic lineage within Sub-Saharan Africa [9], [10] (see Discussion). The other very old haplotypes H19-H21 and H17 are principally Khoe-San, from southern Africa, representing 61% of their chromosomes. Their age can be estimated from the age of mutations marking the corresponding lineages (fig. 2 and table 3; note, however, that when a single branch carries two or more mutations, they are placed in arbitrary order; any of these mutations could be the oldest or the youngest since we cannot determine their relative arrival times, and by convention only the top mutation is considered to be the oldest). The H19-H21 and H17 lineages are separated from the root by mutation 6 timed by *genetree* at 658±163 Ky. They are separated from all other haplotypes that share mutations 1–2 and 3, timed at 503±91 Ky and 464±86 Ky ago, respectively (fig. 2, table 3; note that the order of mutations that occur on the same branch is arbitrary). In turn, more than 75% of Central and Western African chromosomes carry structurally related haplotypes H1 through H11, with the two most frequent sub-Saharan haplotypes, H1 at 34% and H9 at 18%. They occur on two distinct branches H1 and H9, which are split between mutation 3 above and mutations 7, 14 and 21 below, indicating a time of divergence between 300 and 450 Ky ago (table 3). The third separate Eastern clade marked by the haplotype H12 is the most frequent worldwide. H12 and its derived haplotypes account for 73% of the Ethiopian chromosomes, one Kenyan (North East Bantu), one chromosome from Southern Africa (Khoe-San) and all non-African chromosomes. The age of the oldest mutation on this branch (arbitrarily assigned as mutation 7) is also estimated at about 350 Ky (table 3), suggesting divergence time similar to H1 and H9 lineages. The star-like form of H1 and H12 clades points to expansion of the populations carrying these haplotypes, well after the arrival of mutations at sites 25 and 19, i.e. after 151±40 and 85±31 Ky ago, respectively, according to *genetree* age estimates (table 3). Using ρ-statistics [43], [45], equivalent to the approach described by Thomson et al. [47], [48], we obtained values in general similar to those obtained by *genetree*. The exceptions were mutations 7 and 25 (table 3), “defining” branches leading to haplotypes H1 and H12. This can be explained by the fact that *genetree* takes into account full data [42], whereas ρ-statistics only considers the tree information from branches below the mutation in question [45], [62]. Therefore, ρ-statistics estimates may be influenced by local “branch-specific” effects, such as demographic expansions (around H1 and H12) and large differences in branch lengths separating lineages (e.g. ages of mutations 25 and 7). In turn, the ρ-statistics estimate of the age of the mutation 7 is much older than that obtained by *genetree*, reflecting a relative excess of mutations observed along the H12 branch.

**Table 3:** *Genetree* and ρ-statistics time estimates of mutations marking MTMRC8 segment history (figs. 1 and 2) based on 111 sub-Saharan African chromosomes and a Lebanese haplotype 23 chromosome.

	Time estimates in Ky (± S.D.)	
Method	*genetree*	ρ-statistics
TMRCA	745±180	931±290
mut 6	656±145	784±249
mut 3	464±85	532±163
mut 7	336±82	899±373
mut 14	299±85	503±291
mut 21	243±64	200±147
mut 25	151±40	47±24
mut 36	155±55	228±162
mut 19	85±31	78±48

The geographic partition of distinct *MTMR8* lineages between southern, western/central and eastern African populations (fig. 1), with two thirds of the corresponding haplotypes remaining population and region specific, is reminiscent of an ancient stratification of sub-Saharan populations, captured in our data by the population amalgamation test [58] (*p* = 0.025; table 2). One third of these haplotypes are regionally shared, reflecting within Africa gene flow at different historical periods [19]–[22], [67], [68]. Non-standard population demography (i.e. other than non-subdivided constant size population), such as a demographic bottleneck, population subdivision with limited migration and/or population growth, are known to differentially affect estimates of the population mutation parameter Θ. Three distinct estimates of Θ differ in the *MTMR8* data from sub-Saharan Africans: Θ*_П_* = 5.03, from the average number of pairwise differences, Θ*_S_* = 6.45, from the number of segregating sites (table 2; table S3) and Θ*_ML_* = 7.19, a maximum likelihood estimate assuming constant population size model. By computer simulations we explored the effect of different demographic scenarios on the relative values of these estimates. Population bottlenecks reduced all three estimates of mutation rate parameters similarly with respect to the input values. When populations are subdivided and the gene flow becomes increasingly restricted, down to the migration rate 4*Nm* = 0.04, it is the Θ*_П_* that increases the most followed by Θ*_S_*, both characterized by a large variance between individual simulations, whereas Θ*_ML_* increases only slightly. This is in contrast to what we observe here, i.e. Θ*_П_*<Θ*_S_*<Θ*_ML_*. However, the differential effect of population subdivision on the three estimates of Θ disappears, even if the populations were previously kept subdivided, after 500 generations of panmixia; in this scenario, all estimates tend to converge to the input value. Interestingly, a five-fold increase in population size 1500 generations ago has no marked effect on Θ*_П_*, but it doubles Θ*_S_* and triples Θ*_ML_*. In a simulation experiment combining population subdivision with moderate gene flow (4*Nm* = 2) and population growth from 7 000 to 20 000 over the last 15 000 generations and assuming (input) Θ-value of 7.5, we reproduced the three observed Θ estimates: Θ*_П_* = 5.39<Θ*_S_* = 6.33<Θ*_ML_* = 7.20 (i.e. Θ*_ML_* was estimated assuming constant population size). While the simulations do not provide true proof, they do show that both population subdivision and subsequent population growth can shape *MTMR8* diversity in ways consistent with the data, thereby offering a plausible explanation. A moderate increase in population size in Africa is suggested by the shape of the western/central and eastern branches of the network (fig. 1), by the coalescent analysis (fig. 2) and is also consistent with a slightly negative Tajima's *D* (table 2).

## Discussion

### Oldest tree branches belong to Khoe-San

Excluding H22, the oldest lineages H19–H21 and H17 belong to Khoe-San from South-Africa and Namibia, consistent with Khoe-San representing the oldest of all extant populations [68]-[73]. Our results are in line with earlier evidence from uniparentally transmitted markers [24], [74]–[77] and from microsatellite data [20]. Yet, while mtDNA and Y-chromosome studies estimate the oldest Khoe-San lineages at 140 Ky ago [24], [74], [78], our analysis finds the *MTMR8* Khoe-San lineages much older, originating before 300 Ky, possibly around 500 Ky ago and presumably even earlier. This is consistent with observations from a simple inspection of the data: the network structure, branch lengths as well as contrasting age estimates of the mutation 7 (fig. 2 and table 3). The estimates of Khoe-San population divergence, at around 150 Ky ago [71] (calibrated based on human chimpanzee separation at 7.6 My ago), are indeed expected to be much younger than the sequence divergence of the contributing lineages. On the other hand, the same authors approximated African-Eurasian divergence at 55-62 Ky ago, i.e. at almost half the 100 – 120 Ky ago estimated by Li and Durbin [72]. The latter dates are consistent with the archeological evidence of the presence of modern humans in the Near East at around 100 Ky ago [79]–[81] and raise the possibility that Khoe-San divergence estimated to have occurred ∼2.5 times earlier [71] could thus be much older than 150 Ky, closer to the age of their lineages reported here (see however [67], [82]).

### Population bottleneck and sub-Saharan population structure preceding out-of-Africa migration

The intriguing feature of the *MTMR8* tree is the presence of the long lineage of haplotype H12 shared by most Ethiopians, one Kenyan, one Khoe-San and all non-Africans (figs. 1 and 2). Such patterns have been seen before. In the tree derived from *PDHA1* there is a long branch separating the bulk of sub-Saharan-African haplotypes from non-Africans and a small subset of Africans [65]. As in the case of *MTMR8*, this suggests that non-Africans emerged from a subset of Africans living in relative isolation for a substantial length of time consistent with the idea that the out-of-Africa bottleneck started in Africa before the exodus [6], [10]. Note that what we observe is not a local *MTMR8* effect. However, the effect of this bottleneck may be enhanced in this locus due to a selection sweep that seems to have affected 5.4 Mb of the subcentromeric region, including *MTMR8* and 13 other genes [32], [33]. Indeed, our results of the neutrality tests (table 2) are significant in non-Africans and thus consistent with a selection sweep hypothesis. The outcome of the *H* test of Fay & Wu [60] is of special interest, with *H* = −1.84 (*p* = 0.005) for non-Africans, which increased to *H* = −9.44 (*p*<0.001) when non-Africans and Ethiopians were analyzed (fig. 2 and table 3). Outside sub-Saharan Africa we find 46 H12 chromosomes, two differing by single mutations in Europe (H14 and H24) and one chromosome from Lebanon, H23. In Africans the H12 derived lineages are also characterized by low diversity, nine H12 and four H12–derived chromosomes, but the haplotype diversity of H12 branch haplotypes in Africa is significantly higher than all other continental samples taken together (*p* = 0.03 Fisher exact test). And this, in spite of the fact that our Ethiopian sample partly represents non-African chromosomes due to effects of the Eurasian gene-flow on Ethiopian diversity, thus diluting its African component [20], [35], [83], [84]. In Africans we find a haplotype H16 derived from H12 by two mutations. Specifically, H16 is found both in Ethiopians and Khoe-San, two populations known to share deep paternal lineages [67], [68], [85]. Our resulting hypothesis is that a population bottleneck preceded the out of Africa migration and subsequent range expansion, because, at that time, sub-Saharan Africans were stratified and different populations evolved in isolation for an extended period of time. This is plausible, especially through part of the Middle and Late Pleistocene [86], when climatic conditions were conducive or even forced geographic isolation [87]–[89]. Interestingly, all non-African descendants, for both mtDNA and Y-chromosome uniparentally transmitted lines, each share one and the same close African ancestor [90]. This alone provides evidence of a period of isolation in a small single founding group, during which all other founding lineages were lost by genetic drift [82]. If so, the geographic partitioning of genetic diversity we observe should be essentially due to the partitioning of old variants, rather than to the accumulation of novel, population-specific variation, which would reflect recent population history. In *MTMR8*, about two thirds of the haplotypes from each of the three geographic poles (south, west/central or east) represent distinct old lineages (table 1, fig. 1). A similar situation was observed at other X-linked loci and uniparentally inherited markers [6], [10], [23], [24], [75], [82], in spite of recent gene flow affecting several loci to a different extent. To confirm that our observations are not due to a stochastic fluke, limited to a short 11 Kb DNA fragment, we also analysed haplotypes of a 380 Kb segment spanning *MTMR8* locus using the data of HapMap3 populations. The resulting haplotypes' network (fig. S1) is consistent with our earlier findings. It shows a tripartite split between two separate African clusters (only two, in the absence of Central and South-African populations in HapMap3 collection) and one non-African as in the figure 1. Li and Durbin [72] observed an increase in the effective population size in Africa between 200 and 60 Ky ago, which they interpreted as the effect of population fragmentation with reduced migration. Our simulation experiments, while unable to prove this scenario of a complex African population structure before range expansion outside Africa, support its plausibility.

Nevertheless, it is important to note that historical inferences from studies of the X-linked loci may differ from these based on autosomal record. The effective population size of the X-chromosomes is smaller than that of the autosomes and X-chromosomes spend only one third of the time in males and two third in females, where they recombine. Male and female demography may differ, due to different migration patterns of males and females, to patrilocality or matrilocality, to polygamy, and other phenomena changing the female-to-male ratio. As a result, X-chromosomes diversity, as compared to autosomal loci, cannot be simply accounted for by a three-to-four ratio between X-chromosomes and autosomes population sizes [51], [91], [92]. Interestingly, during the out-of-Africa bottleneck, the X-chromosome diversity appears to have been disproportionally reduced relative to the rest of the nuclear genome [93]. Whether this was due to natural selection or to demographic effects as revealed here, it shows how important studies of the X-chromosome diversity (often excluded from genome-wide diversity analyses) are to understand human evolutionary history.

### Archaic admixture

The Biaka haplotype H22 does not share any derived alleles with the remaining haplotypes and is absent outside Africa. Only this haplotype has the alternative allele at our mutation 6 (fig. 1 and table 1) thus driving the *MTMR8* TMRCA back in time prior to the divergence of human and Neandertal lineages. Similar, structurally distinct haplotypes, representing African-only lineages were observed by others. For example, haplotype P in the *CMAH* locus on chromosome 6p21.32 is seen in two copies in Biaka Pygmies out of 132 analyzed chromosomes. It carries 16 derived alleles that are not shared with all other haplotypes carrying a different subset of 39 derived sites. Out of the 56 *CMAH* polymorphisms only one derived allele is shared between P and another haplotype C3 found in the same Biaka population [16]. In the left portion of the *CD209* locus with its 57 segregating sites, 17 derived alleles are exclusive to three rare African haplotypes while the remaining 40 alleles occur on other chromosomes. These three distinct haplotypes are found in San from Namibia (2 copies), in Bantu speakers from Gabon and from South Africa (5), in Yoruba's from Nigeria (3) and Mandenka from Senegal (2) [94]. In turn, in a DNA segment from Xp21.1 haplotype A carries again 6 derived alleles that are exclusive to this haplotype found in only two copies in Mbuti Pygmies and absent on other haplotypes found on the majority of chromosomes [15]. In *CMAH*, *CDC209* and Xp21.1 segment gene trees these rare ancient haplotypes have driven the corresponding TMRCAs above 2 My ago, artificially elongating tree branches due to the presence of derived alleles not shared with all remaining worldwide haplotypes. Other examples of similarly distinct African haplotypes were also found in the *4qMB179*, *13qMG107* and *18qMB60* regions on chromosomes 4q, 13q and 18q, respectively [9]. In the light of our results and examples above, our earlier interpretation of the greater genetic diversity in Africa being partly due to the introgression from an archaic population [6], [10], [36] is gaining more support recently [15], [17], [18], [95]. There is the possibility that these haplotypes are not archaic and have been kept intact for such great amounts of time simply due to the stochastic process. Interestingly, based on HGDP polymorphisms [38] H22 extends over 800 Kb before “joining” new alleles common to other sub-Saharan samples (HT16 in Table S5). It is noteworthy that H19 haplotype (HT17 in Table S5) does not differ from Neandertal and Denisova haplotype over the whole length of the extended haplotype (1971 Kb), confirming its greater antiquity and also consistent with its age estimates.

### Uncertainty in time estimates

Age estimates play an important role in our analysis by providing a temporal framework that allows the integration of results of other genetic studies as well as evolutionary events inferred from the genetic analysis with the paleontological, archaeological and palaeoclimatic context. The ρ-statistic used here is equivalent to the one in Thompson *et al.* (2000) which is considered as a good estimator [48]. Yet, simulations indicated that, albeit infrequently, it has a tendency to underestimate the true value [46], [48]. Uncertainty also comes from genetic estimates such as the substitution rates and the generation times. In present-day hunter-gatherer societies generation time is estimated to be approximately 32 and 26 years for males and females, respectively [96], [97], which leads to an average generation interval of ∼28 years for chromosome X. Here we used 25 years per generation as a phylogenetic average [7] and the same 25 years generation span to convert time into years in the *MTMRC8* tree (table 3). As a consequence of using the same generation estimate (∼25 years) in both calculations, we end up with a homogenous clock with respect to substitutions per year along the entire length of human and chimpanzee branches. This clock not only ignores any changes in generation time but also the possibility of a relative rate slowdown on the human branch [98]. While using 25 rather than 28 years per generation may be justified because of the long evolutionary depth of the human chromosome X lineages [99], it means that our times in fig. 2 and table 3 are possibly underestimates. This effect will be even more pronounced if our phylogenetic substitution rate were overestimated [7], [100], as suggested by recent determinations of the substitution rate directly in human pedigrees [101]-[103]. On the other hand, there are good reasons to believe that these recent pedigree estimates may be too low [104]. Our substitution rate, recalculated from its autosomal equivalent assuming α = 3.3 [102], [104] is ∼1.65×10^−8^ per bp per generation. This figure is very close to the estimates of 1.3×10^−8^ and 1.8×10^−8^ from human Mendelian disease frequencies by Lynch [105] and Kondrashov [106], respectively, and to a recent estimate of 1.4 – 2.3×10^−8^, obtained from a different approach that avoids phylogenetic calibration [104]. Nonetheless we recognize that our ages are likely to be underestimates. On the other hand, our rate was calibrated assuming human-chimpanzee sequence divergence of 7.5 My [7], [49], [107]. Using 6 My instead, as in many earlier studies, would increase our rate estimate by 20% and lower our time estimates by the same factor (see internal left side scale in fig. 2 and table S4). Importantly, such a shift of the time scale would not affect our results and conclusions that point to a longer evolutionary time frame for human evolution, considering a possibility that contemporary humans originated by amalgamation of lineages from different populations that were separated by years of independent evolution.

### Conclusions

The analysis of the *MTMR8* segment diversity and its population tree provided new insights into the evolutionary history of sub-Saharan Africans and demographic events preceding the out of Africa migration. Consistent with earlier studies we found the oldest *MTMR8* lineages among Khoe-San from South-Africa. Worth noting a unique Biaka haplotype branched off directly from the root of the *MTMR8* haplotype tree and may represent a trace of archaic admixture within Africa. Inferred ancient population stratification and the age of the separately evolving lineages deduced from *MTMR8* evolution may imply an older dating of our species, older in fact than the earliest fossils of anatomically modern humans. Additionally, our data indicate that at the time of out of Africa migration sub-Saharan Africans were subdivided for a substantial amount of time. The *MTMR8* segment carries signature of a selection sweep that most likely started prior to the exodus. Such selection events could additionally potentiate the effect of the out-of-Africa demographic bottleneck by reducing the genetic diversity of non-African X chromosomes. Because of its characteristics this subcentromeric region of the X-chromosomes appears particularly interesting to study sub-Saharan populations' history and warrants more detailed analysis using full sequence data.

### Web Resources

Arlequin software, http://cmpg.unibe.ch/software/arlequin3/; Network software, http://www.fluxus-engineering.com/; Program *genetree*, http://www.stats.ox.ac.uk/~griff/software.html; Program msHot, http://home.uchicago.edu/~rhudson1/source/mksamples.html; HGDP data, http://www.cephb.fr/en/hgdp/; HapMap3 data, http://hapmap.ncbi.nlm.nih.gov/.

## Supporting Information

Figure S1 Network of MTMR8 extended haplotypes in HapMap3 populations [39]. The analyzed region extends over 380 Kb (between sequence positions 63312040 to 63693104) and includes 40 SNPs. A total of 53 haplotypes were observed in 1180 X-chromosomes. Africans: LWK, MKK, YRI, and non-Africans: CEU, TSI, JPT, GIH, CHD. Haplotype frequencies are proportional to the surface of the circle (or to its single colored segment within a population group).
(JPG)

Material S1 Supplementary Material.
(DOCX)

Table S1 Samples and their Geographic/Ethnic Origin.
(DOCX)

Table S2 PCR Primer Sequences.
(DOCX)

Table S3 Summary Statistics of Populations Samples. Abbreviations: *n* – number of chromosomes; S – number of segregating sites (SNPs); *k* – number of haplotypes; *G* – gene (haplotype) diversity; (1-*G*) – haplotype homozygosity; Θ – estimator of population mutation rate 4*Nµ* (*N* – effective population size; *μ* - mutation rate per DNA segment per generation); Θ*π* – estimate from nucleotide diversity [108]; Θ*s* – estimate from the number of segregating sites [63]; Θ*_H_* – estimate from frequency of the derived alleles [60]; Θ*_ML_* – maximum likelihood estimate by *genetree* [42]; Θ*_k_* - estimate from the number of haplotypes [55]; Θ*_G_* – estimator from haplotype diversity [109], [110]
(DOCX)

Table S4 Time estimates (Ky ± S.D.) marking the MTMRC8 segment history (figs. 1 and 2) based on phylogenetic calibration of the mutation rate using human-chimpanzee divergence of 6 My.
(DOCX)

Table S5 Extended haplotypes of a subset of our Sub-Saharan samples from HGDP project that were used in our study (Table S1) and for which genome-wide genotypes were available at http://www.cephb.fr/en/hgdp/ [38]. Two positions (A>G8 and T>C36 of the haplotype) whose derived alleles are shared with Neandertals and Denisovans are separated by 1585 Kb, whereas the distance between the same A>G8 and the leftmost C>T24 in the Biaka HT16 (H22 in Fig.1) is 812 Kb. The polymorphism C>T13, with derived T highlighted in red corresponds to the mutation 16 in the network in Fig.1 of the main text. The correspondence between the extended haplotypes below and the Fig.1 network haplotypes are on the right.
(DOCX)
