# Supplementary figures and images for: X-Linked MTMR8 Diversity and Evolutionary History of Sub-Saharan Populations
Source: PLoS One. 2013 Nov 25;8(11):e80710. doi: 10.1371/journal.pone.0080710 (PMC3839994; doi:10.1371/journal.pone.0080710)

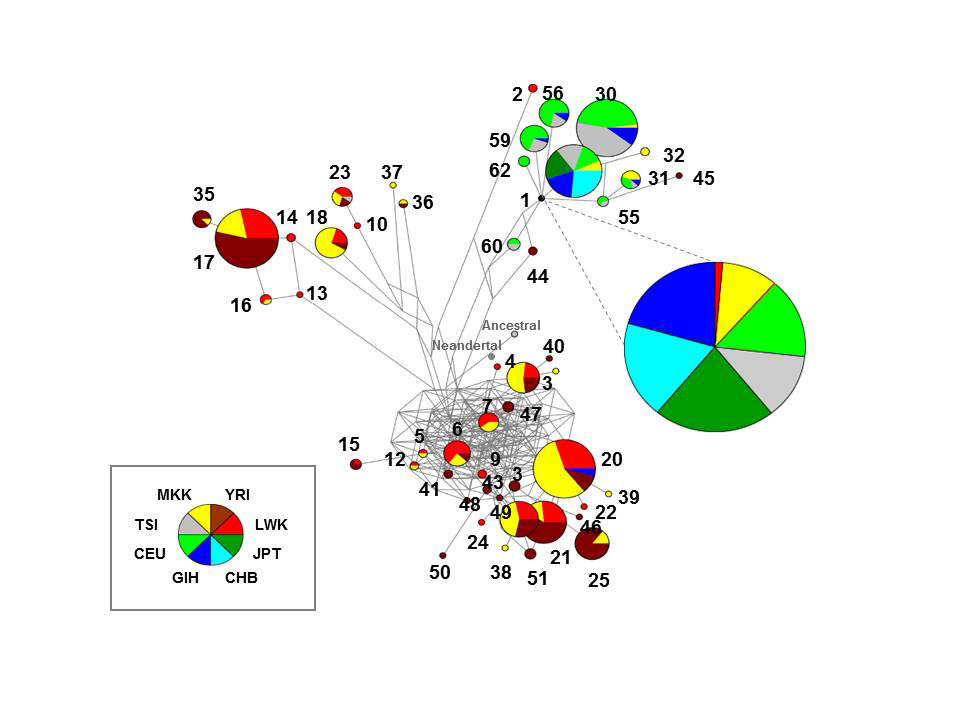

Supplement: Figure S1 — Network of MTMR8 extended haplotypes in HapMap3 populations [39]. The analyzed region extends over 380 Kb (between sequence positions 63312040 to 63693104) and includes 40 SNPs. A total of 53 haplotypes were observed in 1180 X-chromosomes. Africans: LWK, MKK, YRI, and non-Africans: CEU, TSI, JPT, GIH, CHD. Haplotype frequencies are proportional to the surface of the circle (or to its single colored segment within a population group). (JPG) [file pone.0080710.s001.jpg]
