# Supplementary material for: X-Linked MTMR8 Diversity and Evolutionary History of Sub-Saharan Populations
Source: PLoS One. 2013 Nov 25;8(11):e80710. doi: 10.1371/journal.pone.0080710 (PMC3839994; doi:10.1371/journal.pone.0080710)
Supplement: Material S1 — Supplementary Material. (DOCX) [file pone.0080710.s002.docx]

**SUPPLEMENTARY MATERIAL**

**X-linked *MTMR8* diversity and evolutionary history of Sub-Saharan populations.**

Damian Labuda, ^1, 2^ Vania Yotova, ^1^ Jean-François Lefebvre,^1^ Claudia Moreau,^1^ Gerd Utermann,^3^ Scott M. Williams.^4^

^1^ Centre de Recherche, CHU Sainte-Justine, Université de Montréal, Montréal, Québec, Canada

^2^ Département de Pédiatrie, Université de Montréal, Montréal, Québec, Canada

^3^ Department of Medical Genetics, Molecular and Clinical Pharmacology, Medical University of Innsbruck, Austria

^4^ Department of Genetics, Geisel School of Medicine, Dartmouth College, Hanover, NH, USA**Abbreviations :**

*n* – number of chromosomes

S – number of segregating sites (SNPs)

*k* – number of haplotypes

*G* – gene (haplotype) diversity

(1-*G*) – haplotype homozygosity

Θ – estimator of population mutation rate 4*Nµ* (*N* – effective population size; *µ* - mutation rate per DNA segment per generation)

Θ*π* – estimate from nucleotide diversity ([Tajima 1983](#_ENREF_13))

Θ*s* – estimate from the number of segregating sites ([Watterson 1975](#_ENREF_15))

Θ*_H_* – estimate from frequency of the derived alleles ([Fay, Wu 2000](#_ENREF_6))

Θ*_ML_* – maximum likelihood estimate by *genetree* ([Griffiths, Tavare 1994](#_ENREF_8))

Θ*_k_* - estimate from the number of haplotypes ([Ewens 1972](#_ENREF_4))

Θ*_G_* – estimator from haplotype diversity ([Zouros 1979](#_ENREF_18); [Chakraborty, Weiss 1991](#_ENREF_3))

Neutrality tests:

*D* - Tajima’s *D* statistic ([Tajima 1989](#_ENREF_14))

*Fs* – Fu’s *Fs* statistic ([Fu 1997](#_ENREF_7))

*H* – Fay and Wu’s *H* statistics ([Fay, Wu 2000](#_ENREF_6); [Zeng et al. 2006](#_ENREF_17))

Ewens- Watterson homozygosity test ([Watterson 1978](#_ENREF_16))

Slatkin exact test ([Slatkin 1994](#_ENREF_11); [Slatkin 1996](#_ENREF_12))

Chakraborty population amalgamation test ([Chakraborty 1990](#_ENREF_2))

Estimations were obtained using Arlequin V. 3.0 package ([Excoffier, Laval, Schneider 2005](#_ENREF_5)), DnaSP V.5 ([Librado, Rozas 2009](#_ENREF_10)) and *genetree* ([Griffiths, Tavare 1994](#_ENREF_8))

**References**

Altshuler, DM, RA Gibbs, L Peltonen, et al. 2010. Integrating common and rare genetic variation in diverse human populations. Nature 467:52-58.

Chakraborty, R. 1990. Mitochondrial DNA polymorphism reveals hidden heterogeneity within some Asian populations. Am J Hum Genet 47:87-94.

Chakraborty, R, KM Weiss. 1991. Genetic variation of the mitochondrial DNA genome in American Indians is at mutation-drift equilibrium. Am J Phys Anthropol 86:497-506.

Ewens, WJ. 1972. The sampling theory of selectively neutral alleles. Theor Popul Biol 3:87-112.

Excoffier, L, G Laval, S Schneider. 2005. Arlequin ver. 3.0: An integrated software package for population genetics data analysis. Evolutionary Bioinformatics Online 1:47-50.

Fay, JC, CI Wu. 2000. Hitchhiking under positive Darwinian selection. Genetics 155:1405-1413.

Fu, YX. 1997. Statistical tests of neutrality of mutations against population growth, hitchhiking and background selection. Genetics 147:915-925.

Griffiths, RC, S Tavare. 1994. Sampling theory for neutral alleles in a varying environment. Philos Trans R Soc Lond B Biol Sci 344:403-410.

Li, JZ, DM Absher, H Tang, et al. 2008. Worldwide human relationships inferred from genome-wide patterns of variation. Science 319:1100-1104.

Librado, P, J Rozas. 2009. DnaSP v5: a software for comprehensive analysis of DNA polymorphism data. Bioinformatics 25:1451-1452.

Slatkin, M. 1994. An exact test for neutrality based on the Ewens sampling distribution. Genet Res 64:71-74.

Slatkin, M. 1996. A correction to the exact test based on the Ewens sampling distribution. Genet Res 68:259-260.

Tajima, F. 1983. Evolutionary relationship of DNA sequences in finite populations. Genetics 105:437-460.

Tajima, F. 1989. Statistical method for testing the neutral mutation hypothesis by DNA polymorphism. Genetics 123:585-595.

Watterson, GA. 1975. On the number of segregating sites in genetical models without recombination. Theor Popul Biol 7:256-276.

Watterson, GA. 1978. The Homozygosity Test of Neutrality. Genetics 88:405-417.

Zeng, K, YX Fu, S Shi, CI Wu. 2006. Statistical tests for detecting positive selection by utilizing high-frequency variants. Genetics 174:1431-1439.

Zouros, E. 1979. Mutation rates, population sizes and amounts of electrophoretic variation of enzyme loci in natural populations. Genetics 92:623-646.
