# Supplementary material for: X-Linked MTMR8 Diversity and Evolutionary History of Sub-Saharan Populations
Source: PLoS One. 2013 Nov 25;8(11):e80710. doi: 10.1371/journal.pone.0080710 (PMC3839994; doi:10.1371/journal.pone.0080710)
Supplement: Table S1 — Samples and their Geographic/Ethnic Origin. (DOCX) [file pone.0080710.s003.docx]

| **Region** | **Population Country** | | **Sample name** | **Sex** |
| --- | --- | --- | --- | --- |
|  |  |  |  |  |
| **Sub-Saharan Africa** | Bantu North East | Kenya | HGDP01406 | m |
| **n=111** | n=10 |  | HGDP01411 | m |
|  |  |  | HGDP01415 | m |
|  |  |  | HGDP01418 | m |
|  |  |  | HGDP01405 | m |
|  |  |  | HGDP01412 | m |
|  |  |  | HGDP01408 | m |
|  |  |  | HGDP01413 | m |
|  |  |  | HGDP01417 | m |
|  |  |  | HGDP01416 | m |
|  |  |  |  |  |
|  | Biaka Pygmies | Central | HGDP00451 | m |
|  | n=13 | African | HGDP00465 | m |
|  |  | Republic | HGDP00466 | m |
|  |  |  | HGDP00469 | m |
|  |  |  | HGDP00477 | m |
|  |  |  | HGDP00479 | m |
|  |  |  | HGDP00470 | m |
|  |  |  | HGDP00472 | m |
|  |  |  | HGDP00473 | m |
|  |  |  | HGDP00475 | m |
|  |  |  | HGDP00461 | m |
|  |  |  | HGDP00464 | m |
|  |  |  | HGDP00448 | m |
|  |  |  |  |  |
|  | Ethiopians | Ethiopia | Eth401: unclassified | m |
|  | n=15 |  | Eth418: Amhara | m |
|  |  |  | Eth419: Amhara | m |
|  |  |  | Eth426: Oromo | m |
|  |  |  | Eth406: Amhara | m |
|  |  |  | Eth412: Gurage | m |
|  |  |  | Eth413: Gurage | m |
|  |  |  | Eth417: Amhara | m |
|  |  |  | Eth422: Amhara | m |
|  |  |  | Eth423: Oromo | m |
|  |  |  | Eth425: Amhara | m |
|  |  |  | Eth430: Amhara | m |
|  |  |  | Eth414: Amhara | m |
|  |  |  | Eth427: Amhara | m |
|  |  |  | Eth404: Amhara | m |
|  |  |  |  |  |
|  | Gabon |  | Gab019 | m |
|  | n=12 |  | Gab085 | m |
|  |  |  | Gab022 | m |
|  |  |  | Gab077 | m |
|  |  |  | Gab101 | m |
|  |  |  | Gab009 | m |
|  |  |  | Gab032 | m |
|  |  |  | Gab050 | m |
|  |  |  | Gab090 | m |
|  |  |  | Gab098 | m |
|  |  |  | Gab073 | m |
|  |  |  | Gab005 | m |
|  |  |  |  |  |
|  | Khoe-San | South-Africa | Khoi08 | m |
|  | n=11 |  | Khoi206 | m |
|  |  |  | Khoi329 | m |
|  |  |  | KhoiB183 | m |
|  |  |  | Khoi110 | m |
|  |  |  | Khoi338 | m |
|  |  |  | KhoiB131 | m |
|  |  |  | KhoiB172 | m |
|  |  |  | KhoiB186 | m |
|  |  |  | KhoiB169 | m |
|  |  |  | Khoi14 | m |
|  |  |  |  |  |
|  | San | Namibia | HGDP00991 | m |
|  | n=7 |  | HGDP01032 | m |
|  |  |  | HGDP00987 | m |
|  |  |  | HGDP01029 | m |
|  |  |  | HGDP01036 | m |
|  |  |  | HGDP0988 | m |
|  |  |  | HGDP477 | m |
|  |  |  |  |  |
|  | Mandenka | Senegal | HGDP00907 | m |
|  | n=13 |  | HGDP00911 | m |
|  |  |  | HGDP00919 | m |
|  |  |  | HGDP01199 | m |
|  |  |  | HGDP01284 | m |
|  |  |  | HGDP00908 | m |
|  |  |  | HGDP01202 | m |
|  |  |  | HGDP00904 | m |
|  |  |  | HGDP00913 | m |
|  |  |  | HGDP01200 | m |
|  |  |  | HGDP01283 | m |
|  |  |  | HGDP00906 | m |
|  |  |  | HGDP00912 | m |
|  |  |  |  |  |
|  | Mbuti Pygmies | Democratic | HGDP00449 | m |
|  | n=13 | Republic of | HGDP00450 | m |
|  |  | Congo | HGDP00462 | m |
|  |  |  | HGDP00463 | m |
|  |  |  | HGDP00467 | m |
|  |  |  | HGDP00474 | m |
|  |  |  | HGDP00982 | m |
|  |  |  | HGDP00984 | m |
|  |  |  | HGDP00456 | m |
|  |  |  | HGDP01081 | m |
|  |  |  | HGDP00478 | m |
|  |  |  | HGDP00983 | m |
|  |  |  | HGDP00468 | m |
|  |  |  |  |  |
|  | Yoruba | Nigeria | HGDP00923 | m |
|  | n=15 |  | HGDP00929 | m |
|  |  |  | HGDP00934 | f |
|  |  |  | HGDP00941 | m |
|  |  |  | HGDP00942 | m |
|  |  |  | HGDP00944 | m |
|  |  |  | HGDP00933 | f |
|  |  |  | HGDP00931 | m |
|  |  |  | HGDP00927 | m |
|  |  |  | HGDP00930 | m |
|  |  |  | HGDP00936 | m |
|  |  |  | HGDP00940 | m |
|  |  |  | HGDP00943 | m |
|  |  |  | HGDP00932 | m |
|  |  |  | HGDP00937 | m |
| **Non Africa** | Near East North Africa | Iran | BM44 | f |
| **n=49** | n=12 | Lebanon | BM82 | f |
|  |  | Lebanon | BM108 | f |
|  |  | Egypt | BM81 | f |
|  |  | Egypt | BM154 | f |
|  |  | Morocco | BM25 | f |
|  |  |  |  |  |
|  | Europe | Bulgarian | Lab1 | f |
|  | n=13 | French | Fr1008 | f |
|  |  | French-Canadian | BM88 | f |
|  |  | German | BM55 | f |
|  |  | Italian | It13628 | f |
|  |  | Italian | it 12557 | f |
|  |  | French | Lab8 | m |
|  |  |  |  |  |
|  | South East Asia | China | lab xx | f |
|  | n=14 | Vietnam | BM49 | f |
|  |  | China | BM147 | f |
|  |  | China | chold15 | f |
|  |  | Philippine | BM94 | f |
|  |  | India | BM134 | f |
|  |  | Korea | lab7 | f |
|  |  |  |  |  |
|  | Native Americas | Chipewyans | Nad83 | f |
|  | n=10 | Chipewyans | Nad89 | f |
|  |  | Chipewyans | Oj1271 | f |
|  |  | Maya | JK1617 | f |
|  |  | Maya | JK1583 | f |
