# Supplementary material for: X-Linked MTMR8 Diversity and Evolutionary History of Sub-Saharan Populations
Source: PLoS One. 2013 Nov 25;8(11):e80710. doi: 10.1371/journal.pone.0080710 (PMC3839994; doi:10.1371/journal.pone.0080710)
Supplement: Table S3 — Summary Statistics of Populations Samples. Abbreviations: n – number of chromosomes; S – number of segregating sites (SNPs); k – number of haplotypes; G – gene (haplotype) diversity; (1-G) – haplotype homozygosity; Θ – estimator of population mutation rate 4Nµ (N – effective population size; μ - mutation rate per DNA segment per generation); Θπ – estimate from nucleotide diversity [108]; Θs – estimate from the number of segregating sites [63]; ΘH – estimate from frequency of the derived alleles [60]; ΘML – maximum likelihood estimate by genetree [42]; Θk - estimate from the number of haplotypes [55]; ΘG – estimator from haplotype diversity [109], [110] (DOCX) [file pone.0080710.s005.docx]

| Population | ***n*** | ***S*** | **Θ*s*** | **Θ*_П_*** | **Θ*_H_*** | ***k*** | ***G*** | **Θ*_k_*** | **Θ*_G_*** |
| --- | --- | --- | --- | --- | --- | --- | --- | --- | --- |
| Bantu (Kenya) | 10 | 18 | 6.36 | 4.20 | 5.80 | 7 | 0.87 | 9.02 | 5.39 |
| Biaka | 13 | 12 | 3.87 | 5.21 | 5.80 | 4 | 0.76 | 1.57 | 2.42 |
| Ethiopia | 15 | 15 | 4.61 | 4.25 | 7.18 | 6 | 0.70 | 3.20 | 1.83 |
| Gabon | 12 | 11 | 3.64 | 4.15 | 6.03 | 5 | 0.79 | 2.68 | 2.93 |
| KhoiSan | 18 | 18 | 5.23 | 4.40 | 1.40 | 8 | 0.83 | 4.95 | 3.94 |
| Mandenka | 13 | 8 | 2.58 | 3.21 | 2.97 | 4 | 0.72 | 1.57 | 1.96 |
| M’Buti | 13 | 7 | 2.26 | 1.33 | 7.02 | 5 | 0.53 | 2.49 | 1.28 |
| Yoruba | 17 | 14 | 4.14 | 3.72 | 6.03 | 6 | 0.76 | 2.86 | 2.54 |
| **All Sub-Saharan Africans** | **111** | **34** | **6.45** | **5.03** | **6.13** | **21** | **0.83** | **7.40** | **4.09** |
| Near-East / North Africa | 12 | 1 | 0.33 | 0.17 | 1.84 | 2 | 0.17 | 0.39 | 0.15 |
| South East Asia | 14 | 0 | 0.00 | 0.00 | 0.00 | 1 | 0.00 | 0.00 | 0.00 |
| Europe | 13 | 2 | 0.64 | 0.31 | 0.03 | 3 | 0.29 | 0.89 | 0.31 |
| Native America | 10 | 0 | 0.00 | 0.00 | 0.00 | 1 | 0.00 | 0.00 | 0.00 |
| **All Non-Africans** | **49** | **3** | **0.67** | **0.12** | **1.96** | **4** | **0.12** | **0.84** | **0.10** |
| **Total** | 160 | 36 | 6.37 | 5.80 | 7.50 | 24 | 0.81 | 7.60 | 3.32 |
