# Supplementary material for: X-Linked MTMR8 Diversity and Evolutionary History of Sub-Saharan Populations
Source: PLoS One. 2013 Nov 25;8(11):e80710. doi: 10.1371/journal.pone.0080710 (PMC3839994; doi:10.1371/journal.pone.0080710)
Supplement: Table S4 — Time estimates (Ky ± S.D.) marking the MTMRC8 segment history (figs. 1 and 2) based on phylogenetic calibration of the mutation rate using human-chimpanzee divergence of 6 My. (DOCX) [file pone.0080710.s006.docx]

|  | *genetree* | ρ -statistics |
| --- | --- | --- |
| TMRCA | 596 ± 144 | 745 ± 232 |
| mut 6 | 526 ± 116 | 627 ± 199 |
| mut 3 | 371 ± 69 | 426 ± 130 |
| mut 7 | 269 ± 66 | 719 ± 298 |
| mut 14 | 239 ± 68 | 402 ± 233 |
| mut 21 | 194 ± 51 | 160 ± 118 |
| mut 25 | 121 ± 32 | 38 ± 19 |
| mut 19 | 68 ± 25 | 62 ± 38 |
