# Supplementary material for: X-Linked MTMR8 Diversity and Evolutionary History of Sub-Saharan Populations
Source: PLoS One. 2013 Nov 25;8(11):e80710. doi: 10.1371/journal.pone.0080710 (PMC3839994; doi:10.1371/journal.pone.0080710)
Supplement: Table S5 — Extended haplotypes of a subset of our Sub-Saharan samples from HGDP project that were used in our study (Table S1) and for which genome-wide genotypes were available at http://www.cephb.fr/en/hgdp/ [38]. Two positions (A>G8 and T>C36 of the haplotype) whose derived alleles are shared with Neandertals and Denisovans are separated by 1585 Kb, whereas the distance between the same A>G8 and the leftmost C>T24 in the Biaka HT16 (H22 in Fig.1) is 812 Kb. The polymorphism C>T13, with derived T highlighted in red corresponds to the mutation 16 in the network in Fig.1 of the main text. The correspondence between the extended haplotypes below and the Fig.1 network haplotypes are on the right. (DOCX) [file pone.0080710.s007.docx]

Polymorphic sites of the 1971 Kb haplotype

(corresponding sequence positions are shown below)

Chimp ACCGCTT**A**GGCC**C**CTTTGCTTGCCCTAGGTCTCTG**T**CTGCC

Altai -------**G**---------------------------**C**-----

Denis -------**G**---------------------------**C**-----

Bantu Biaka Mandenka Mbuti San Yoruba(Fig.1)

**HT17 -------G---------------------------C----- 1 (H19)**

**HT16 ---AA--G---------------T--------T-ACT-AT- 3 (H22)**

**HT3 ---AAC-G--------C----A------A------CT---- 1 (H12)**

**(Below is family of H9)**

HT26 ----------T-**T**---------T--G-T-------CT-AT- 1 2

HT18 ---A------T-**T**---------T--G-T----------ATT 1

HT19 ---A------T-**T**---------T--G-T-------CTG--- 1

HT20 ---A------T-**T**---------T--G-T-------CT-AT- 1 1 3 2

HT30 ---A------T-**T**---------T--G-T-------CT---- 1

HT36 ---A------T-**T**---------T--G-T----------AT- 1

**(Families of H1 to H11 except H9)**

HT23 ---A-C-G-----------C---T--------T-A------ 1

HT1 -----C-G-----------C---T-------CT-A--G--- 1

HT4 -----C-G-----------C---T-------CT-AC---TT 1

HT22 -----C-G-----------C---T-------CT-AC----- 1

HT10 ---AAC-G-----------C---T-------CT-A------ 1

HT37 -----C-GA-----C----CC--T-------CT-A---ATT 1

HT7 -----C-GA-----C----C---T--------T-------- 1

HT34 -----C-GAA----C----C--------------AC----- 2

HT35 ---AA--GAA----C----C---T--------T-A------ 1

HT29 ---AA--GAA----C----C---T--------T-AC----T 1

HT11 ---AA--GAA----C-?--C---T--------T-A------ 1 1

HT14 ---AA--GAA----C----CC--T-------CT-AC----- 1

HT21 ---AA--GAA----C----CC--T-------CT-----AT- 1

HT32 ---AA--GAA----C----CC--T-------CT-A---AT- 1

HT5 ---AA--GAA----C----C---T-------CT-AC---TT 3

HT9 ---AA--GAA----C----C---T-------CT-AC----T 2

HT12 ---AA--GAA----C----C---T-------CT-ACT---- 1

HT13 ---AA--GAA----C----C---T-------CT-A------ 3

HT27 ---AA--GAA----C----C---T-------CT-AC----- 2

HT29 ---AA--GAA----C----C---T--------T-AC----T 1

HT11 ---AA--GAA----C-?--C---T--------T-A------ 1 1

HT6 ---AA--GAA--**-**TC----CC--T-------CT-A---AT- 1 4 1 2

HT2 ---AA--GAA---TC----CC--T-------CT-AC----T 1 1

HT8 ---AA--GAA---TC----C---T-------CT-ACTG--- 1

HT15 ---AA--GAA---TC----CC--T-------CT-AC--AT- 1 1

HT24 ---AA--GAA---TC----CC--T-------CTCA--G--T 1

HT25 ---AA--GAA---TC----CC--T-------CT-A---ATT 1 1

HT33 ---AA--GAA---TC----CC--T-------CTCA---ATT 1 1

HT28 ---AA--GAA---TC----CC--T-------CTCA---AT- 1 1

Sequence positions of the polymorphic sites of the haplotypes above

63024078 63053467 63055937 63072861 63165232 63188822 63223967 **63242059** 63283604 63465852 63484882 63553851 **63569307** 63636559 63644888 63689233 63707347 63749154 63787412 63972393 63998301 64041387 64045135 **64053909** 64087388 64190860 64253096 64261813 64280697 64405169 64592435 64596501 64634861 64637612 64733990 **64827053** 64827473 64894437 64912303 64948690 64995453
