# Supplementary material for: X-Linked MTMR8 Diversity and Evolutionary History of Sub-Saharan Populations
Source: PLoS One. 2013 Nov 25;8(11):e80710. doi: 10.1371/journal.pone.0080710 (PMC3839994; doi:10.1371/journal.pone.0080710)
Supplement: Supplementary file 7 [file pone.0080710.s004.docx]

Fragment Name	Primer Sequence (5' --> 3') F	R	Product Length (bp)	chrom X start**	
7	tgaaatcagactgaatccttatacc	ttcatcatctggcctatctgc	577	63481252	intron 5-6<br>63,480,326<br>63,481,649
**exon5**	gccaacattcgcttcagattcatgggcattgagaacatccatgtaatgcggagcagtctg<br>cagaaactcttggaag			63481650	76
8	tcttcattttcatacccccttcc	cattgaaccttgtgattgtgc	563	63481733	
9	ctgtcattaaggctggagtgc	attgggagatgagaaaataggc	573	63482250	
10	agggagtaaatgagacttgtgg	caggttccatttttgtctctcc	645	63482692	
11	ctgggtaggccacctaatgc	caggttccattttcctctctcc	658	63483189	
12	caaattggagagaggaaaatgg	aacattgctgaacagagtgtcc	701	63483819	
13	gagccacacaaaactttgaagg	gcagtgttattgggaaagttagg	770	63484425	
14	cacacatacacaaacctaacttcc	ccagacattcatgttaaatagttacg	756	63485158	
15	agaagggtggtttgtttcaagc	ttaaacatggctctggaagacc	671	63485847	
16	gcagcatgaaacttaaaacagg	tcctacgtgtgcatatcagagc	542	63486476	
17	ttttgctctgatatgcacacg	tccaaagtttaagtccaggtaagg	697	63486992	
18	gttttctacccaccccccttacc	acttcagcttttggattcttgg	696	63487650	intron 4-5<br>63,481,726<br>63,491,399
19	agagcagccttcagtttgtgg	aaaaatcagcaaagggaaaagg	690	63488263	
20	cctggttttgttgactttgtcc	aaggaggtcaggaagtgaagc	715	63488903	
21	ctccagcaaacgaattctcc	taacccatttgcctatttttgc	420	63489437	
1	gccaactaaagcaaaaataggc	gctgggattataggtgtgaacc	481	63489825	
2	ggtggtggttcacacctataatcccagc	cactccactacccttaccaacc	505	63490284	
3	caaagcatagaaagacaaatattgc	aagagatctcatggcctaaagc	548	63490659	
4	ttccttaggctgattttcatgc	acacaccacagtccttttctgg	543	63491134	
**exon 4**	cattacctgaagatctttatgctttttcttataatcccaaatcctcaaaagagatgaggg			63491400	140
5	tcatcaaacaatgcaaataaagg	cctaggcaattaaccagcttcc	684	63491592	intron 3-4<br>63,491,540<br>63,492,780

** Ensemble Release March 2006
